# The NLRP3 Inflammasome Role in the Pathogenesis of Pregnancy Induced Hypertension and Preeclampsia

**DOI:** 10.3390/cells9071642

**Published:** 2020-07-08

**Authors:** Maciej W. Socha, Bartosz Malinowski, Oskar Puk, Mariusz Dubiel, Michał Wiciński

**Affiliations:** 1 Department of Obstetrics, Gynecology and Gynecological Oncology, Faculty of Medicine, Collegium Medicum in Bydgoszcz, Nicolaus Copernicus University, Ujejskiego 75, 85-168 Bydgoszcz, Poland; kikpoloz@cm.umk.pl; 2 Department of Obstetrics and Gynecology, St. Adalbert’s Hospital in Gdańsk, Jana Pawła II 50, 80-462 Gdańsk, Poland; 3 Department of Pharmacology and Therapeutics, Faculty of Medicine, Collegium Medicum in Bydgoszcz, Nicolaus Copernicus University, M. Curie 9, 85-090 Bydgoszcz, Poland; bartosz.malin@gmail.com (B.M.); oskar.trebacz@gmail.com (O.P.); wicinski4@wp.pl (M.W.)

**Keywords:** NLRP3, inflammation, preeclampsia, pregnancy-induced hypertension, HELLP syndrome, immunothrombosis

## Abstract

Pregnancy-induced hypertension and preeclampsia are associated with significant maternal and fetal mortality. A better understanding of these diseases, delineation of molecular pathomechanism, and efficient treatment development are some of the most urgent tasks in obstetrics and gynecology. Recent findings indicate the crucial role of inflammation in the development of hypertension and preeclampsia. Although the mechanism is very complex and needs further explanation, it appears that high levels of cholesterol, urate, and glucose activates NLRP3 inflammasome, which produces IL-1β, IL-18, and gasdermin D. Production of these proinflammatory chemokines is the beginning of a local and general inflammation, which results in sympathetic outflow, angiotensin II production, proteinuria, hemolysis, liver damage, immunothrombosis, and coagulopathy. The NLRP3 inflammasome is a critical complex in the mediation of the inflammatory response, which makes it crucial for the development of pregnancy-induced hypertension and preeclampsia, as well as its complications, such as placental abruption and HELLP (hemolysis, elevated liver enzymes, and low platelets) syndrome. Herein, the presented article delineates molecular mechanisms of these processes, indicating directions of future advance.

## 1. Introduction

Pregnancy-induced hypertension (PIH), defined as a systolic blood pressure > 140 mmHg and/or a diastolic blood pressure > 90 mmHg, complicates approximately 6–10% of all pregnancies, and is related to significant maternal and fetal mortality and the increased risk of placental abruption, cerebrovascular events, organ failure, and disseminated intravascular coagulation [1]. Gestational hypertension most frequently occurs as a component of preeclampsia (PE), defined as de-novo hypertension present after 20 weeks of gestation combined with proteinuria (>300 mg/day), other maternal organ dysfunction, hematological complications, uteroplacental dysfunction, or fetal growth restriction [1]. PE afflicts 2–8% of pregnancies and is one of the three leading causes of maternal morbidity and mortality worldwide [2]. Preeclampsia can be complicated with eclampsia, stroke, abruptio placentae, disseminated intravascular coagulation (DIC), liver hemorrhage/rupture, pulmonary edema, or HELLP syndrome, and the latter is the cause of 83% of deaths in the course of PE [1,2]. Recognizing the gravity of this problem, scientists for decades tried to discover the precise pathomechanism of PE, and introduce efficient therapeutic options. Regarding that pursuit Weel et al. performed immunohistochemical analyses of placental tissues collected from 20 women with PE and 20 healthy controls [3]. Researchers discovered a significantly higher expression of the NOD-like receptor family, pyrin domain-containing protein 3 (NLRP3), and related mediators such as caspase-1, IL-1β, and IL-18 in samples from women with PE compared to controls [3]. Moreover, Xu et al. and Pontillo et al. reported that specific NLRP3 gene polymorphisms are associated with a significantly higher risk of PE development [4,5]. This article aims to delineate the role of NLRP3 in the development of preeclampsia, its symptoms, and complications.

## 2. Role of NLRP3 in PE and PIH

### 2.1. General Information

**Figure 1:**
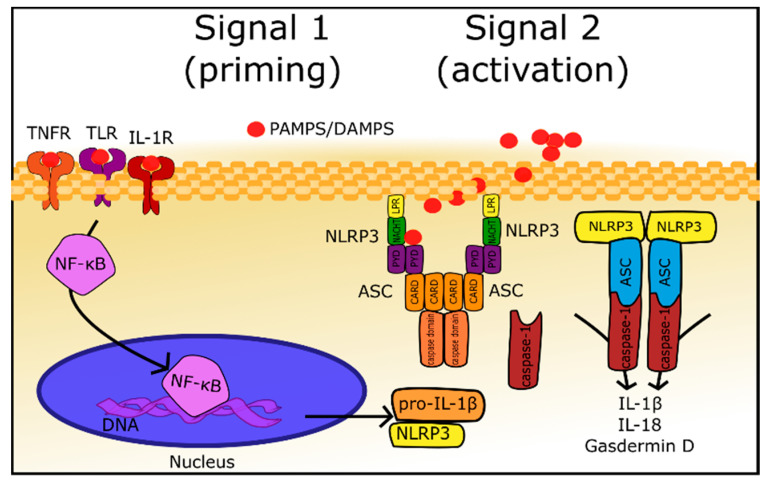
Mechanism of the formation and activation of the NLRP3 inflammasome (detailed description in Section 2.1). **Signal 1** (priming): activation of NF-κB (nuclear factor kappa B) by DAMPs/PAMPs through TNFR (tumor necrosis factor receptor), TLR (Toll-like receptor), and IL-1R (interleukin 1 receptor). NF-κB stimulates the production of NLRP3 and pro-IL-1β. **Signal 2** (activation): DAMPs/PAMPs connects directly to the NLRP3, changes its confirmation and leads to its multimerization and inflammasome formation. Active inflammasome recruits and activates caspase 1, which in turn activates IL-1β, IL-18 and gasdermin D.

The innate immune system is a host’s first line of defense against invading pathogens or environmental irritants; it allows a rapid response to the danger and maintains homeostasis. At the beginning host cells must detect pathogens, which express specific molecules called pathogen-associated molecular patterns (PAMPs) and identify molecules associated with inflammation and cell destruction, which are termed damage-associated molecular patterns (DAMPs). In order to surveil and recognize PAMPs and DAMPs, every cell expresses germline-encoded pattern recognition receptors (PRRs), such as Toll-like receptors (TLRs) and nucleotide-binding domain leucine-rich repeat-containing receptors (NLRs). Among 22 recognized members of the NLR family, NLRP3 (NOD-like receptor family, pyrin domain-containing protein 3) is most extensively investigated, due to its ability to associate with other proteins in large multimeric complexes, called inflammasomes (Figure 1) [6]. Furthermore, inflammasomes, activated by PAMPS and DAMPs, produce inflammatory response mediators, which leads to further activation of the immune system [7]. Considering its structure, which is delineated in Figure 1, the NLRP3 inflammasome consists of NLRP3, ASC, and the cysteine protease precursor procaspase-1 [8]. NLRP3 is composed of C-terminal leucine-rich repeats (LRRs), a central nucleotide-binding and oligomerization domain (NACHT), and an N-terminal pyrin domain (PYD) which is an effector domain. ASC, also termed Pycard, is an abbreviation of an apoptosis-associated speck-like protein containing an N-terminal PYD and a C-terminal caspase recruitment domain (CARD), and the name itself describes its structure. The last element of the NLRP3 inflammasome, cysteine protease precursor procaspase-1, is composed of the CARD and caspase domain. Activation of NLRP3 inflammasome involves two signals, both initiated by DAMPs or PAMPs [6,7,8]. The first one leads to the activation of the nuclear factor κ B (NF-κB) through different receptors such as NLRs, TLRs, IL-1R1, etc. Nuclear factor κ B is a transcription factor that interacts with a vast number of genes, and, among other effects of transcription regulation, it induces the expression of pro-IL-1β and NLRP3. These proteins are not constitutively expressed in the majority of cells, therefore, their concentrations in not-primed cells are insufficient to form inflammasomes [7]. The second signal mechanism is not fully delineated; however, it appears that it involves the direct binding of DAMPs to NLRP3 [7]. Activated NLRP3 multimerizes and interacts with ASC, which is responsible for the recruitment and activation of procaspase-1 into caspase-1 [7,8]. Active caspase-1 transforms pro-IL-1β, pro-IL-18, and gasdermin D (GSDMD) into their active forms (Figure 1) [6,7,8].

### 2.2. NLRP3 as an Inductor of Hypertension

**Figure 2:**
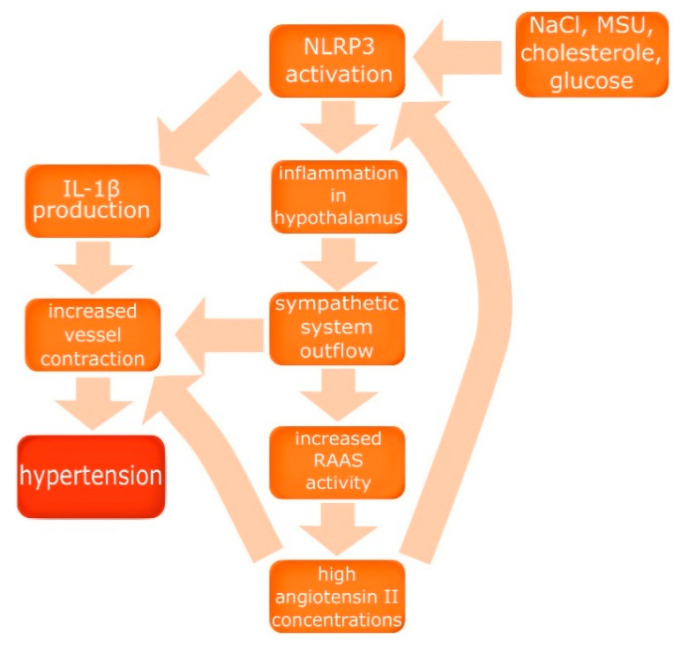
The role of NLRP3 inflammasome in the development of hypertension. Activation of NLRP3 by NaCl, monosodium urate (MSU), or cholesterol induces inflammation in the hypothalamus, leading to sympathetic system outflow, which results in the activation of renin–angiotensin–aldosterone system (RAAS), and angiotensin II leads to further induction of inflammation and NLRP3 activation. IL-1β has a direct impact on arteries.

Many scientists noticed the role of the immune system in the pathomechanism of hypertension, and a vast number of studies indicating inflammation and elevated levels of plasma interleukins among patients with hypertension were analyzed by Krishnan et al. in order to answer the question: are IL-1β and IL-18 mediators or just markers of hypertension [9]? As described above, IL-1β and IL-18 are products of activated NLRP3 inflammasome; therefore, its role in the development of hypertension was investigated [8]. Omi et al. examined 1911 patients (987 with hypertension, 924 controls), discovering that homozygotes of high activity NLRP3 alleles, whose NLRP3 inflammasomes produce more chemokines after stimulation, had a greater risk of hypertension development compared to both heterozygotes and homozygotes of low activity NLRP3 alleles (odds ratio 1.24, *p* = 0.03) [10]. These findings led to conclusion that NLRP3 inflammasome activity has an important role in the development of hypertension. However, some questions still need an answer, such as how exactly the inflammasome is activated in the course of hypertension, are there some specific triggers, and what is the precise mechanism leading to increase of blood pressure? To address these questions other factors, crucial for the development of hypertension, must be considered, such as the renin–angiotensin–aldosterone system (RAAS) and the sympathetic system. Van der Meiraker and Boomsma indicated a connection between these two systems by discovering that the activity of both is increased in hypertension. Moreover, ACE or AT1 inhibitors resulted in a decrease of sympathetic system outflow, despite predictions that lowering blood pressure would increase sympathetic system activity [11]. Torretti reported increased renin excretion and production under sympathetic system stimulation through the activation of β-adrenergic receptors in juxtaglomerular cells, indicating the balance and cooperation of RAAS and the sympathetic system [12] The cooperation between these two systems was very useful in the past, helping to maintain proper blood pressure and sodium homeostasis in case of low sodium intake, which afflicted humans for thousands of years [12]. To assess the impact of the present salt abundance on the human body, Rust and Eckmekcioglu investigated the role of salt intake in pathogenesis of hypertension [13]. Researchers described the potential of increased natrium chloride concentrations to induce inflammation in the brain, leading to sympathetic system outflow and as a result, the increase of renin concentrations, all contributing to blood pressure elevation [13]. Analysis of these findings and reported NLRP3 activation by NaCl prompt Liu et al. to conclude that high salt intake induces NLRP3 mediated inflammation in the brain, in the hypothalamic paraventricular nucleus, which leads to sympathoexcitation and RAAS activation [8]. Furthermore, Platten et al. show a significant reduction of inflammatory lesions, the amount of Th-1 and Th-17 lymphocytes, and an increase of the number of Treg lymphocytes in a rat experimental autoimmune encephalitis model after treatment with ACE or AT-1 inhibitors, indicating a notable role of RAAS in inflammation induction and maintenance [14]. This indicates that angiotensin II production is induced by inflammation while inflammation is induced by angiotensin, and the linking factor, which closes that loop, appears to be NLRP3 inflammasome. In order to confirm that hypothesis and delineate the role of NLRP3 in the development of hypertension in preeclampsia, Shirasuna et al. induced PE in pregnant wild type mice, NLRP3 knock-out mice (NLRP3 −/−), and ASC knock-out mice (asc −/−) [15]. Researchers divided female mice aged 8–12 weeks into seven groups of approximately four individuals each: wild type vehicle, wild type 500 ng/kg, wild type 1500 ng/kg, NLRP3 −/− 500 ng/kg, NLRP3 −/− 1500 ng/kg, asc −/− 500 ng/kg, and asc −/− 1500 ng/kg. All animals had an osmotic minipump implanted into a dorsal space on the 10th day of gestation, and those minipumps administered continuously 500 or 1500 ng/kg of angiotensin II daily for eight days. Angiotensin II elevated systolic blood pressure (SBP) in a dosage-dependent fashion [15]. However, in both NLRP3 −/− and asc −/− groups SBP was significantly lower in comparison to the wild type group, and this effect was considerably more pronounced among NLRP3 knock-out mice than ASC knock-out mice, indicating the substantial role of NLRP3 inflammasome in the development of hypertension in preeclampsia [15]. However, the increase of Ang II and the sympathetic outflow are not all processes participating in the development of NLRP3 inflammasome-induced hypertension. Krishnan et al. described the contractile response of arteries directly after exposition to IL-1β, and augmented contraction after activation of α1-adrenergic receptors in an IL-1β rich environment [9]. Furthermore, IL-1β and IL-18 are associated with the increase of pro-oxidant enzyme expression in blood vessels and the increase of intima-media complex thickness in carotid arteries [9]. Considering the aforementioned data induction of NLRP3 i.e., by high concentrations of NaCl, induces inflammation in the paraventricular nucleus of the hypothalamus, leading to sympathetic outflow, which results in the activation of RAAS, and angiotensin II leads to further induction of inflammation and NLRP3 activation, which closes the cycle of hypertension pathomechanism. Additionally, IL-1β, a product of the activated NLRP3 inflammasome, has a direct impact on arteries inducing and facilitating contraction. The role of NLRP3 inflammasome in the development of hypertension has been summarized in Figure 2.

### 2.3. Role of NLRP3 in Kidney Injury

Along with hypertension, proteinuria is a major sign of PE, and it indicates kidney injury. Kwon et al. assessed the urine of 45 patients with confirmed lupus nephritis; 36 of them had no or mild tubulointerstitial inflammation (TI), and nine had moderate to severe TI [16]. Results show a significant correlation between proteinuria and moderate to severe TI (odds ratio [OR] 3.166, 95% confidence interval (95% CI) 1.145–8.757, *p* = 0.026) describing proteinuria as a diagnostic and prognostic parameter of tubulointerstitial inflammation [16]. Furthermore, Li and Zhuo reported that tubulointerstitial inflammation is initiated by Ang II through the activation of NF-κB, which, as a transcription factor, increases the expression of proinflammatory chemokines [17]. Therefore, Ang II appears to be harmful to kidneys and, as it was described in the previous section, its production is stimulated by NLRP3 inflammasome, and thus this mechanism may contribute to proteinuria in PE; however, this hypothesis needed confirmation. Grande et al. conducted a study, which shed more light on this process, indicating that Ang II activates NF-κB through transforming the growth factor β (TGFβ), and the production of reactive oxygen species (ROS) [18]. Afterwards, NF-κB stimulates macrophage infiltration of renal interstitium and the apoptosis of renal tubular cells by increasing the production of proinflammatory chemokines [18]. In order to investigate if NLRP3 inflammasome takes part in the development of tubulointerstitial inflammation and injury Tashiro et al. assessed kidney biopsy specimens of 28 patients with TI [19]. Researchers discovered that IL-β1 was elevated in all samples and positively correlated with the severity of the tubulointerstitial injury. Moreover, there was a positive correlation of NLRP3 mRNA with the severity of TI as well, which indicates that NLRP3 inflammasome is associated with TI and might contribute to its development [19]. As was described above, NLRP3 inflammasome activates not only IL-β1 and IL-18 but also gasdermin D, and Li et al. reported that gasdermin D can lead to kidney injury through the induction of renal tubular cell pyroptosis in the course of treatment with cisplatin [20]. This information implicates that gasdermin D is a possible inductor of renal cell destruction as a consequence of NLRP3 inflammasome activation and TI induction by Ang II. Involvement of the immunological system in the development of PIH associated proteinuria was also investigated by Neßelhut et al. who compared the urinary protein profile of 21 healthy males, 25 healthy females, 64 patients with an uncomplicated pregnancy, and 110 hypertensive pregnant women [21]. Researchers detected that Tamm–Horsfall’s glycoprotein was present in the urine of healthy individuals, but was significantly decreased or absent in the urine of 83% of hypertensive pregnant women. Based on studies indicating that Tamm–Horsfall’s glycoprotein is, in fact, uromodulin and has a significant affinity to the IL-1, Neβelhut et al. hypothesized that the immunological system has a substantial role in the development of PIH and PE. Therefore, the reduction of the urine’s Tamm–Horsfall glycoprotein concentration is a result of its immunosuppressive activity and association with IL-1 [21]. Considering the aforementioned data, the activation of NF-κB and NLRP3 through Ang II, leading to inflammation, renal tubular cell pyroptosis, and proteinuria is a pathomechanism of kidney injury in hypertension, i.e., in PE or PIH. However, it appears that TI is not the only cause of kidney injury and proteinuria in PE. Yamamoto et al. examined the urine of 34 preeclamptic patients, dividing protein profiles into four patterns: low molecular weight (L) pattern (tubular damage), high MW (H) pattern (glomerular damage), high and low MW (HL) pattern, and middle MW (M) pattern. The incidences of the HL, H, L, and M patterns were 26.5%, 14.7%, 11.8%, and 47.1% respectively [22]. Furthermore, Kaltenbach et al. performed a similar study investigating 107 pregnant women with PE, additionally dividing patients into different groups based on their MAP: 80–100 mmHg, 100–120 mmHg, and 120–200 mmHg. Results showed that 47% of all patients had mixed patterns, indicating both glomerular and tubular pathology. Interestingly, the glomerular pattern prevalence in 80–100 mmHg, 100–120 mmHg, 120–200 mmHg groups was 3%, 7%, and 12%, respectively, indicating the correlation of glomerulopathy with blood pressure [23]. According to this data kidney injury in PE occurs not only due to TI, but also glomerulopathy which could be a result of coagulopathy and/or hemolysis associated with NLRP3 activity, and these processes are described in following sections of this article.

### 2.4. NLRP3 Impact on the Coagulation System

**Figure 3:**
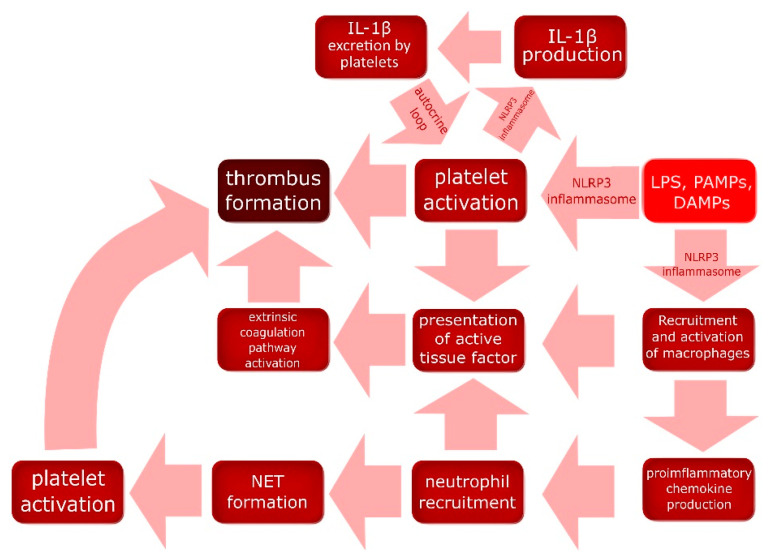
NLRP3 inflammasome’s role in immunothrombosis. NLRP3 inflammasome activated by DAMPs and PAMPs activates platelets and leukocytes which in turn have the potential to activate the extrinsic pathway of the coagulation cascade, which leads to thrombus formation. Platelet activation is further enhanced though an IL-1β-mediated autocrine-paracrine loop. DAMPs—damage-associated molecular patterns; PAMPs—pathogen-associated molecular patterns; LPS—lipopolysaccharide; NET—neutrophil extracellular trap.

Coagulation is a complex process, the initiation of which involves many different mediators and cells, including those forming the immune system. Interestingly, during an infection the role of the coagulation system is not to prevent bleeding but to embed pathogens and facilitate their destruction in a process called immunothrombosis (Figure 3) [24]. In their review Engelmann and Massberg indicate that lipopolysaccharide (LPS) and other PAMPs associated with infection, and DAMPs associated with inflammation can directly activate and induce platelet aggregation, due to the expression of TLRs (TLR2 and TLR4) on cell surfaces. Moreover, LPS, other PAMPs, and DAMPs can activate a yet undefined subset of macrophages through i.e., TLRs, inducing proinflammatory chemokine production. Inflammation leads to the recruitment of more monocytes, which differentiates into macrophages, and other white blood cells, inter alia neutrophils, which releases neutrophils extracellular traps (NETs) composed of DNA associated with histones, and histones H3 and H4 can activate platelets [24]. Furthermore, activated macrophages, neutrophils, and platelets present an active tissue factor, which activates the extrinsic pathway of coagulation by interaction with the factor VIIa, which activates the coagulation factor X, and the active factor X induces the production of thrombin. The role of immunothrombosis is to trap pathogens, i.e., bacteria, and prevent them from spreading with the blood, which facilitates the destruction of pathogens and limits the inflammatory response to small loci instead of the whole body. Interestingly, the hemolytic abilities of some bacteria have been known for decades, and it appears that it is a counteraction against immunothrombosis [24]. Murthy et al. conducted a study aiming to determine whether NLRP3 is involved in the formation of a thrombus through platelet activation and aggregation. Researchers assessed the expression of NLRP3 and ASC in non-activated and collagen-activated platelets using immunofluorescence staining, which has shown significant up-regulation of NLRP3 and ASC in activated platelets [25]. Moreover, a western blot revealed a notable increase in the amount of active IL-1β in a collagen-activated platelets specimen in comparison to resting platelets. Furthermore, platelet aggregometry and a flow chamber with a collagen-coated surface showed a significant reduction of platelet activation and aggregation in the blood of NLRP3 knock-out mice in comparison to wild type mice [25]. The same tests were performed using blood samples of healthy humans. Platelets were isolated and pretreated for 30 min with a specific inhibitor against NLRP3 or against caspase-1. Platelets of both groups showed a significant reduction of activation and aggregation in comparison to samples without inhibitors, proving that NLRP3 inflammasome is crucial for thrombus formation [25]. Brown et al. experimented on platelets isolated from human blood samples, indicating that IL-1β and IL-1α stimulates pro- IL-1β production in platelets [26]. Flow cytometry showed 1ILR1 expression on the platelets’ surface, and the recombinant IL1R antagonist abolished the aforementioned effect of IL-1, leading to the conclusion that this receptor is responsible for signal transduction. Moreover, as it was described above, platelets through NLRP3 and caspase-1 can activate pro-IL-1β. Brown et al. claimed the existence of an IL-1β autocrine loop, which is a part of the immunothrombosis process [26]. In relation to the previous section, thrombosis in renal microvessels can lead to glomerulopathy and the subsequent development of proteinuria [27]. These data confirm the crucial role of NLRP3 in the mechanism of immunothrobosis. Interestingly, platelets constitutively express NLRP3, therefore, thrombocytes do not need a priming signal for the activation of the inflammasome, which highlights its importance in thrombus formation [28]. Considering the correlation of PE with NLRP3 and all described pathomechanisms, this inflammasome appears to play a significant role in development of PE associated coagulopathy. The mechanism of immunothrobosis is summarized in Figure 3.

### 2.5. NLRP3 as an Inductor of Placental Abruption

Preeclampsia is a risk factor of placental abruption, a major obstetric complication, with fetus mortality around 49.5% and mother mortality in the range of 4–8% [29,30]. For decades scientists struggled to delineate the pathomechanism of placental abruption and develop more efficient therapies. Nath et al. performed a histological examination of placenta samples of 160 patients with placental abruption, and 176 patients with no evidence of placental abruption. All women delivered at the gestation of 20 weeks or longer, and samples were taken right after the parturition [31]. These samples were evaluated to determine the presence of chorioamnionitis, defined as the presence of inflammatory infiltrates of neutrophils at two or more sites of the chorionic plate and extraplacental membranes. The odds ratio for chorioamnionitis for women with placental abruption in comparison to controls was 3.6 (95% CI 1.7 to 10.5), and 2.8 (95% CI 1.3 to 6.1) for preterm and term gestation groups, respectively, indicating the participation of an immunology system in placental abruption process [31]. Moreover, analysis of the placental tissue of 20 normotensive pregnant women and 20 patients with severe PE, performed by Weel et al., revealed a significant increase of NLRP3, caspase-1, and IL-1β expression [3]. This data indicates the occurrence of NLRP3 inflammasome mediated inflammation in the placentae of women with PE. More interesting findings were shown by Braila et al. in their histological study of placental abruption, where edema and inflammation were detected in both the uterus and the placenta [32]. Moreover, researchers discovered thrombosis and coagulant imbalance in the uterus and placental infarctions in the examined samples. Furthermore, myometrium presented a vascular network poor in small blood vessels [32]. These findings can be explained by the aforementioned placental inflammation and NLRP3-associated coagulopathy, and Major et al. reported the production of soluble fms-like tyrosine kinase-1 (sFlt-1) by monocytes stimulated by activated platelets in the blood of women with PE [33]. Soluble fms-like tyrosine kinase-1 is a vascular endothelial growth factor (VEGF) inhibitor, and due to this property, it can impede angiogenesis. Therefore, it can be hypothesized that placental NRLP3-mediated inflammation leads to immunothrombosis, and the monocyte-platelet aggregates produce sFlt-1, which inhibits angiogenesis by reducing the levels of active VEGF. Both coagulopathy and angiogenesis inhibition possibly contribute to placental incapacity and placental abruption. However, this hypothesis needs confirmation.

### 2.6. NLRP3 as an Inductor of HELLP Syndrome

**Figure 4:**
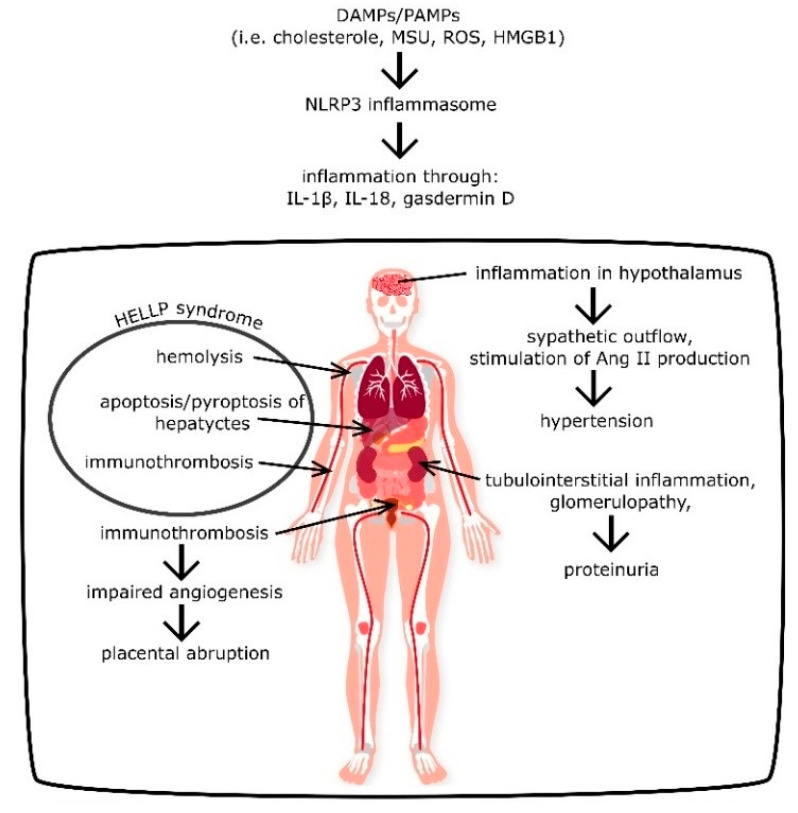
Pathomechanism of pregnancy-induced hypertension (PIH) and preeclampsia (PE) development by the NLRP3 inflammasome. DAMPs—damage associated molecular patterns, PAMPs—patogen associated molecular patterns, MSU—monosodium urate, ROS—reactive oxygen species, HMGB1—high-mobility group box 1 protein.

HELLP is an abbreviation of hemolysis, elevated liver enzymes, and low platelets. HELLP syndrome is a grave obstetric complication with severe maternal mortality and afflicts 10–20% of women with preeclampsia [34]. Abildgaard and Heimdal delineated the pathomechanism of HELLP syndrome, indicating a significant role of thrombotic microangiopathy in the development of this disease [35]. The pathomechanism of coagulopathy and thrombosis in small vessels among women with PE, as well as the role of NRLP3 in that process, is described in Section 2.4. Red blood cells are fragmented as they pass through vessels with fibrin strands of thrombus, which results in microangiopathic hemolytic anemia [35]. Moreover, hem released from fragmented erythrocytes acts as a DAMP, and therefore stimulates NLRP3 inflammasome activation and the further induction of immunothrombosis and erythrocyte destruction [36]. Furthermore, Mustafa and Bano discovered that bilirubin, a product of hem metabolism, deactivates cystatin, an inhibitor of cathepsin, which increases the activity of the latter [37]. Cathepsin is a protease that initiates apoptosis of hepatocytes through caspase-3 and caspase-9, leading to liver damage. Therefore, bilirubin from fragmented erythrocytes can indirectly induce liver parenchyma destruction [37,38]. Moreover, cathepsin and thrombin directly cleaves the complement component 5 (C5) to C5a, facilitating the formation of the membrane attack complex (C5b-9), and C5b-9 damages cell membranes inducing apoptosis or necrosis [39]. To confirm association of C5b-9 with PE, Burwick et al. examined blood and urine samples of 25 women with severe PE, 25 women with chronic hypertension, and 25 healthy controls, discovering significantly higher concentrations of C5a and C5b-9 in the plasma of women from the first two groups in comparison to the controls [40]. Furthermore, researchers detected high concentrations of C5b-9 in the urine samples of 94% of women with severe preeclampsia, and none in other groups, indicating the participation of C5b-9 and C5a in the pathomechanism of PE [40]. The membrane attack complex directly haemolyses red blood cells in the intravascular hemolysis process, contributing to DMAPs production and spread of inflammation [41]. If the inflammatory response occurs in the liver, activated NLRP3 inflammasome leads to the production of gasdermin D and pyroptosis of hepatocytes, which contribute to elevation in liver enzyme concentrations [42]. Interestingly, placentas with thrombotic and ischaemic alterations, as it was described in Section 2.5, show higher expression of death receptor Fas ligand (FasL), which can induce apoptosis of hepatocytes, since hepatocytes maintain constitutive expression of Fas [35]. Therefore, this could be an another component of the liver damage in HELLP syndrome among preeclamptic women [43]. Considering the aforementioned data, HELLP syndrome is most likely a result of NLRP3-mediated inflammation (Figure 4).

## 3. NLRP3 Activators in PE and PIH

The role of NLRP3 inflammasome as a risk factor of PIH and PE, and its crucial part in the pathomechanism of these diseases appears to be quite well established. However, the cause of primary NLRP3 inflammasome activation still needs an explanation. Assessment of blood samples performed by Stødle et al. shed some light on that process by revealing elevated plasma concentrations of monosodium urate (MSU) and cholesterol among women with PE in comparison to healthy controls [44]. Moreover, MSU and cholesterol showed a positive correlation with plasma concentrations of high sensitivity C-reactive protein and soluble sFlt-1 (a marker of PE), indicating the association of MSU and cholesterol with inflammation and PE [44]. To confirm if MSU induces an inflammatory response in preeclampsia Matias et al. assessed monocytes isolated from blood samples of 23 women with PE and 23 normotensive pregnant women [45]. Monocytes of women with PE showed significantly higher expression of NLRP3, caspase-1, IL-1β, and IL-18 mRNA. Interestingly, monocyte stimulation with MSU resulted in the increased expression of NLRP3, caspase-1, and IL-18 in samples of PE women but not in samples of controls. Moreover, longer incubation with MSU increased the expression of caspase-1 and IL-1β in a dosage-dependent fashion in the monocytes of non-pregnant healthy women. Furthermore, this effect was abolished by glibenclamide, an NLRP3 inhibitor [45]. Another, similarly designed, study confirmed that NLRP3 is activated by cholesterol, ROS, MSU, hyaluronan, and high glucose concentrations [45,46,47,48,49]. These findings led to the creation of the NLRP3 crystal-induced activation theory, where MSU, alum, silica, asbestos, β-amyloid, cholesterol crystals, and calcium crystals, induces NLRP3 inflammasome activation [6,45]. Although the exact mechanism is not yet comprehended, this theory is vital for PE research, because it explains why high BMI, which is usually connected with hypercholesterolemia, is a significant risk factor for PE development [1]. Therefore, crystal-induced NLRP3 inflammasome activation theory can facilitate the development of effective prophylaxis and treatment of preeclampsia.

## 4. Outstanding Issues and Directions of Future Development

Immunological and hormonal balance is very complex and fragile, mainly during pregnancy, when many changes occur. One of them is an alteration of cholesterol concentration, which typically decreases at the beginning of pregnancy achieving nadir in the second month, and significantly increases above concentrations from before pregnancy, with the zenith in the month of parturition [50]. As was described above, cholesterol is an activator of the NLRP3 inflammasome, and thus physiological for pregnancy, increase of its concentration could be a primer of preeclampsia development. However, cholesterol level elevation is typical for pregnancy, but PE afflicts only 2–8% of pregnancies. Therefore, there is another factor, which makes some women more susceptible to DAMPs like cholesterol, and/or which facilitates a greater inflammatory response and/or impairs the self-restraint ability of the immunological system. Interestingly Stødle et al. revealed no difference of NLRP3 expression in placenta samples of healthy and preeclamptic patients. However, if PE was complicated with IUGR, placentae exhibited increased levels of IL-1β, a product of NLRP3 inflammasome [44]. Similar expressions of NLRP3 exclude the previous priming, but higher IL-1β production could be an effect of NLRP3 polymorphism and the presence of its more active forms. This finding indicates that on the top of the general inflammatory response, there are local inflammations, which could be a reason for the variable PE spectrum. Despite many unknowns in the pathomechanism of PE development studies proving the crucial role of NLRP3 in that process and delineating DAMPs, which can activate the inflammasome can be a source of great change in the therapy and prophylaxis of preeclampsia. For example, by monitoring and lowering levels of cholesterol and monosodium urate, or through the introduction of new pharmacotherapy guidelines. Interestingly, even glucose in high concentrations activates NLRP3 inflammasome in trophoblasts, and a recent study showed that metformin inhibits NLRP3 through the AMPK/mTOR pathway. Therefore this drug could be used as a PE prevention among pregnant patients with DM2 (diabetes mellitus type 2) or gestational diabetes [49,51]. Moreover, researchers investigated the potential of different NLRP3 inhibitors, which, if safe for fetuses, would significantly decrease PE morbidity and mortality, which is the future of obstetrics [1,52].

## 5. Conclusions

NLRP3 plays a crucial role in the development of PIH and PE, leading to the occurrence of hypertension, through sympathetic outflow and RAAS activation, to proteinuria, through tubulointerstitial inflammation and glomerulopathy, to placental abruption, through immunothrombosis, and to the development of HELLP syndrome, through immunothrobosis, cytokine-mediated hemolysis, and the induction of hepatocyte apoptosis and pyroptosis. The NLRP3 inflammasome can be activated by high concentrations of cholesterol, glucose, MSU, ROS, etc., although other factors, like genetic susceptibility, appear to be important for excessive activation of the inflammasome and the development of PIH and PE. Therefore, inhibitors of NLRP3 could be a very effective treatment of pregnancy-induced hypertension and preeclampsia.
